# Dopamine in nucleus accumbens: salience modulation in latent inhibition and overshadowing

**DOI:** 10.1177/0269881110389211

**Published:** 2011-12

**Authors:** AJD Nelson, KE Thur, CA Marsden, HJ Cassaday

**Affiliations:** Institute of Neuroscience, Schools of Psychology and Biomedical Sciences, University of Nottingham, UK.

**Keywords:** Dopamine, latent inhibition, nucleus accumbens, overshadowing

## Abstract

Latent inhibition (LI) is demonstrated when non-reinforced pre-exposure to a to-be-conditioned stimulus retards later learning. Learning is similarly retarded in overshadowing, in this case using the relative intensity of competing cues to manipulate associability. Electrolytic/excitotoxic lesions to shell accumbens (NAc) and systemic amphetamine both reliably abolish LI. Here a conditioned emotional response procedure was used to demonstrate LI and overshadowing and to examine the role of dopamine (DA) within NAc. Experiment 1 showed that LI but not overshadowing was abolished by systemic amphetamine (1.0 mg/kg i.p.). In Experiment 2, 6-hydroxydopamine (6-OHDA) was used to lesion DA terminals within NAc: both shell- and core- (plus shell-)lesioned rats showed normal LI and overshadowing. Experiment 3 compared the effects of amphetamine microinjected at shell and core coordinates prior to conditioning: LI, but not overshadowing, was abolished by 10.0 but not 5.0 µg/side amphetamine injected in core but not shell NAc. These results suggest that the abolition of LI produced by NAc shell lesions is not readily reproduced by regionally restricted DA depletion within NAc; core rather than shell NAc mediates amphetamine-induced abolition of LI; overshadowing is modulated by different neural substrates.

## Introduction

Past experience with a stimulus in the form of pre-exposure without consequences normally reduces the level of associative learning that the pre-exposed stimulus can support (Lubow and Moore, 1959). In a variety of procedures, this latent inhibition (LI) effect is reliably abolished by treatment with low-dose amphetamine in both humans (Gray et al., 1992; Kumari et al., 1999) and rats (Crider et al., 1982; Solomon et al., 1981; Weiner et al., 1981, 1984, 1987, 1988). LI is similarly abolished in cases of schizophrenia (Baruch et al., 1998; Serra et al., 2001) and after electrolytic and excitotoxic lesions to the shell subfield of the dopaminergic structure the nucleus accumbens (NAc) (Tai et al., 1995; Weiner, 2003; Weiner et al., 1996, 1999). Abolished LI is seen as reflecting ‘hyperassociability’, manifest as increased conditioning to a stimulus that would normally be treated as irrelevant. Because aberrant processing of stimulus salience has been hypothesized to contribute to the cognitive abnormalities of schizophrenia (Bleuler, 1911; Kapur, 2003, 2004), LI has gained widespread acceptance as a model for schizophrenic attention disorder (for reviews see Gray et al., 1991, 1997, 1999; Weiner 1990, 2003; Weiner and Feldon, 1997; Weiner and Arad, 2009).

With respect to the underlying psychological mechanisms, LI is one of a wider set of procedures that can be used to examine the substrates of hyperassociability. Overshadowing procedures use the relative intensity of competing cues to manipulate associability: normally a relatively more intense stimulus acquires associative strength at the expense of a relatively less intense stimulus. Similar to LI, when overshadowing is abolished hyperassociability is manifest as conditioning to a stimulus that would normally be of low salience (cf. Kapur, 2003, 2004). Similar to LI, overshadowing has been reported to be abolished by treatment with amphetamine (Oades et al., 1987; O’Tuathaigh and Moran, 2002, 2004; O’Tuathaigh et al., 2003) and hippocampal lesions (Schmajuk et al., 1983). Thus, the substrates responsible for this effect could well be equivalent to those mediating LI (Cassaday, 2010; Cassaday and Moran, 2010). However, as yet, the neuroanatomical basis of overshadowing is unclear, e.g., some studies have reported no effect on overshadowing of lesions to hippocampus (Garrud et al., 1984; Good and Macphail, 1994) and NAc (Horsley et al., 2008).

In the present study, we used a conditioned emotional response (CER) procedure to demonstrate LI and overshadowing with equivalent experimental parameters, varying only the essential procedural details necessary to demonstrate LI versus overshadowing, to examine the effects of three experimental manipulations. Experiment 1 tested the effects of systemic amphetamine. Experiment 2 tested the effects of 6-hydroxydopamine (6-OHDA) injected within the shell and core NAc subregions to produce differential depletion in medial shell and core. Experiments 3A and 3B examined the effect of amphetamine micro-injected at coordinates adapted from the Experiment 2 lesion study.

Studies using electrolytic and neurotoxic lesions have shown different effects of shell and core NAc lesions, with shell disrupting LI and core or whole NAc sparing and enhancing LI depending on parametric conditions, yielding and not yielding LI in controls, respectively (Schiller et al., 2006; Weiner, 2003; Weiner et al., 1996, 1999). Where there is no LI in controls, DA depletion produced by 6-OHDA has previously been reported to enhance LI (Joseph et al., 2000). With the LI parameters used here, selected to demonstrate amphetamine-induced abolition of LI and compare effects on overshadowing, the equivalent DA depletion would be expected to spare LI. However, the DA depletions made by Joseph et al. (2000) were centred on core NAc. The effects of varying the placement of 6-OHDA injection within NAc, to allow some dissociation of shell and core subfields, have yet to be examined. There are also *in vivo* dialysis and voltammetry studies which suggest a dissociable role of DA within shell versus core NAc in LI: specifically stimulus pre-exposure is associated with a reduction of DA in the shell compared with that seen on presentation of the non pre-exposed stimulus (Jeanblanc et al., 2002; Murphy et al., 2001). The reported effects of amphetamine injected in NAc have been inconsistent. This manipulation has been reported to disrupt (Joseph et al., 2000; Solomon and Staton, 1982) or to spare LI (Ellenbroek et al., 1997; Killcross and Robbins, 1993).

Thus, the three experimental manipulations used in the present study were selected with two aims: (1) to address the role of shell and core NAc with respect to the mediation of effects on LI by comparing the effects of 6-OHDA and amphetamine injected at different coordinates within NAc; (2) to systematically compare the effects of the same experimental manipulations on overshadowing, to determine whether the two phenomena have common neural substrates (Cassaday and Moran, 2010; Kapur, 2003, 2004). Based on the above literature, the predictions for the present study are as follows: (1) the systemic amphetamine treatment used in Experiment 1 will disrupt LI and overshadowing; (2) 6-OHDA injected in core NAc will spare LI in Experiment 2 (as this was conducted with parameters yielding LI in controls; cf. Joseph et al., 2000); (3) since pre-exposure reduces DA in the shell, DA depletion in NAc shell should similarly spare LI (when conducted with parameters yielding LI in controls; see Jeanblanc et al. 2002; Murphy et al. 2001); (4) amphetamine injected in NAc core will disrupt LI.

## Methods

### Subjects

Experimentally naive adult male Wistar rats (Charles River, UK) were caged in pairs on a 12 h:12 h light/dark cycle with food and water *ad libitum*. On arrival, rats were handled for approximately 10 min per day for 1 week. Procedures were carried out in accordance with the United Kingdom (UK) Animals Scientific Procedures Act 1986, Project Licence number: PPL 40/2648 (Experiment 1); and PPL 40/3163 (Experiments 2 and 3). The UK Act ensures full compliance with the ‘Principles of laboratory animal care’ (NIH publication No. 86-23, revised 1985).

### Apparatus

Six identical fully automated conditioning chambers, housed within sound-attenuating cases containing ventilation fans (Cambridge Cognition, Cambridge, UK), were used in Experiments 1–3. Each of the inner conditioning chambers consisted of a plain steel box (25 cm × 25 cm × 22 cm high) with a Plexiglas door (19 cm × 27 cm) at the front. The floor was a shock grid with steel bars 1 cm apart and 1 cm above the lip of a 7 cm deep sawdust tray. Mounted in one wall were three stimulus lights and a waterspout. The spout was 5 cm above the floor and connected to a lickometer supplied by a pump. Licks were registered by breaking the photo beam within the spout, which also triggered water delivery of 0.05 mL per lick. The waterspout was illuminated when water was available. A loudspeaker for the presentation of auditory stimuli was set in the roof. A 5 s flashing light, provided by the three wall-mounted stimulus lights and the house light flashing both on (0.5 s) and off (0.5 s) served as the conditioned stimulus (CS) for control and pre-exposed animals (there was no other background illumination). In the overshadowing condition, the 5 s light CS was presented in compound with a 5 s mixed frequency noise set at 85 dB (including background noise from the fans). Scrambled footshock of 1 s duration and 1 mA intensity provided the unconditioned stimulus (UCS). This was delivered through the grid floor by a constant current shock generator (pulsed voltage: output square wave 10 ms on, 80 ms off, 370 V peak under no load conditions; MISAC Systems, Newbury, UK). Stimulus control and data collection was by an Acorn Archimedes RISC computer programmed in Basic with additional interfacing using an Arachnid extension (Cambridge Cognition).

### Procedure

Water deprivation was introduced 1 day prior to shaping. Thereafter, the animals received 1 h and 15 min of *ad libitum* access to water in their home cage in addition to water in the experimental chambers. The stages of the conditioned emotional response (CER) procedure used in Experiments 1–3 were as follows.

#### Pre-training

Rats were shaped for 1 day until all drank from the waterspout and individually assigned to a conditioning box for the duration of the experiment. Rats subsequently drank in the experimental chamber for 15 min each day (timed from first lick). The drinking spout was illuminated throughout, but no other stimuli were presented in this phase. Latency to first lick was measured to determine readiness to drink in the experimental context. The 10 days pre-training used in Experiment 1 were subsequently shortened to 5 days in Experiments 2 and 3 for the time-limited surgical studies.

#### Pre-exposure

Animals were placed in the chambers where the pre-exposed animals received 30 5 s flashing light CS presentations with an average inter-stimulus interval of 60 s. The control and overshadowing animals were confined to the chambers for an identical period of time without receiving the light CS presentations. Water was not available within the chamber and the waterspout was not illuminated during the pre-exposure session.

#### Conditioning

Conditioning was conducted on the day following pre-exposure. No water was available within the chamber and the waterspout was not illuminated. There were two conditioning trials in which the UCS footshock was delivered following termination of the CS. The first pairing of CS and UCS was presented after 5 min had elapsed, and the second pairing was 5 min after the first, followed by a further 5 min left in the apparatus. For the non-pre-exposed and pre-exposed animals the flashing light served as the CS. In the overshadowing condition, the light CS was presented in compound with the salient noise stimulus. In the absence of drinking, there were no behavioural measures to record.

#### Reshaping

On the day following conditioning, animals were reshaped following the same procedure as in pre-training sessions. This was in order to re-establish drinking after conditioning. Reshaping also provided measures of conditioning to the box context (latency to first lick).

#### Light test

On the day following reshape, the animals were placed in the conditioning chambers and underwent an extinction test to the light CS. Water was available throughout the test and the waterspout was illuminated. Once the animals had made 50 licks, the light CS was presented for 15 min. Excluding the time to first lick, the latency to make 50 licks in the absence of the CS (the A period) provided a measure of any individual variation in baseline lick responding. This was compared with the time taken to complete 50 licks following CS onset (B period) in a suppression ratio (A/(A + B)) to assess the level of conditioning to the light CS, adjusted for any individual variation in drink rate.

#### Noise test

On the day following the light test, the level of conditioning to the overshadowing CS in the overshadowing group was assessed in an extinction test, conducted in exactly the same manner except that the noise CS was presented. The non-pre-exposed group served as the control group in the noise test.

### Experiment 1: Systemic amphetamine administration

#### Drug administration

Seventy-one rats (mean weight of 221 g, in the range 187–244 g) were allocated to be treated with systemic amphetamine (*n = *36) or vehicle (*n = *35), administered (i.p.) 15 min prior to the pre-exposure and conditioning stages of LI. D-amphetamine sulphate (Sigma, Poole, UK) was dissolved in physiological saline to an injection volume of 1.0 mL/kg. The 1.0 mg/kg dose was calculated as the salt. Control animals received an equivalent volume of saline. The reshape and test sessions were conducted drug-free.

### Experiment 2: Shell and core 6-OHDA lesions

#### Surgical procedures

One hundred and eight rats (mean weight 211 g, in the range 180–277 g) underwent surgery, for two replications of the tests of LI and overshadowing. In total, 36 rats were injected with 6-OHDA at shell coordinates and 36 rats were injected at core coordinates, 36 rats received sham lesions (18 were vehicle-injected at the core coordinates and 18 were vehicle-injected at the shell coordinates). Neurochemical assay was the final arbiter of lesion group (as described in the following). One core-injected rat from the second replication died postoperatively.

In order to protect noradrenergic terminals, animals received subcutaneous administration of the noradrenalin (NA) reuptake inhibitor desipramine (20.0 mg/kg) 40 min prior to surgery. Anaesthesia was induced by isoflurane (4%) in a N_2_O/O_2_ (1 : 2, v/v) mixture and maintained thereafter with isoflurane (1–2%). Stereotaxic surgery was conducted with the incisor bar set at −3.3 mm below the intra-aural line. A craniotomy was performed with a 1 mm hand drill (to make a hole of approximate diameter 1 mm) and the dura was cut to expose the cortex. In Experiment 2, rats received bilateral infusions of 6-OHDA or vehicle into either NAcc core or medial shell at the following stereotaxic coordinates from bregma: core at AP + 1.6 mm, ML ±1.8 mm, DV −6.8 mm; medial shell at AP + 1.3 mm, ML± 0.8 mm, DV −6.4 mm and 7.0 mm; one infusion at each DV coordinate (Paxinos and Watson, 2005). DV coordinates were taken from dura. Infusions were made via a 31 gauge stainless steel injector attached by polythene tubing to a 1 µL Hamilton syringe. 6-OHDA hydrobromide (24.0 mg/mL as salt dissolved in vehicle; Sigma, UK) or vehicle (0.9% saline/ascorbic acid 0.01% w/v) was infused manually over 2 min on each side in a volume of 0.5 µL (core) or as two infusions of 0.25 µL (medial shell). The injectors were left *in situ* for 5 min to allow absorption of the bolus and to minimize spread of the toxin. Control animals were injected with the vehicle at shell or core coordinates and otherwise treated identically. Rimadyl (0.03 mL s.c.) provided post-operative analgesia. Animals were allowed a 5–10 day recovery before the commencement of behavioural testing (the recovery period varied somewhat as the lesions were conducted over 5 days).

#### Neurochemical assay

**Figure 1.**
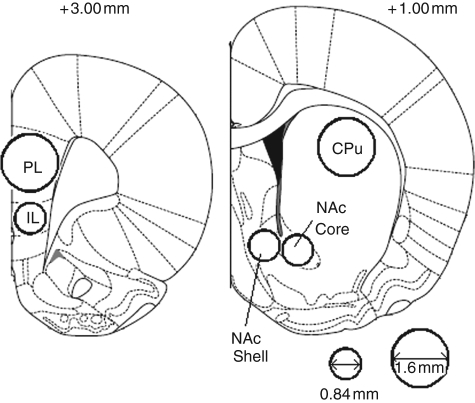
Forebrain regions dissected for postmortem neurochemical analysis. Regions of interest were dissected by pushing micropunch needles of 0.84 or 1.6 mm diameter into the posterior face of the coronal slices as indicated. (Adapted from Paxinos and Watson (2005) *The Rat Brain in Streretaxic Coordinates, 5th edition* with permission from Elsevier). Numbers indicate distance from bregma in millimetres.

Following the completion of behavioural testing, rats were humanely killed by dislocation of the neck and decapitated. The brains were removed rapidly and dissected on a cold tray. A 2 mm coronal slice of brain containing the ventral and dorsal striatum and a separate 2 mm slice containing the medial prefrontal cortex were made using a chilled brain matrix (Harvard Instruments, USA). The brain samples were then immediately frozen on dry ice and stored at −80°C. Subsequently, a 0.84 mm diameter stainless steel micropunch was used to remove samples of tissue from the following (left and right) brain regions: core NAc, medial shell NAc, and infralimbic cortex. A 1.6 mm diameter stainless steel micropunch was used to remove sample tissue from the caudate putamen and prelimbic cortex (Figure 1). Tissue punch samples were stored in 1.5 mL Eppendorf tubes and frozen at −80°C.

Neurotransmitter levels in the samples were determined by high-pressure liquid chromatography (HPLC) with electrochemical detection. The tissue samples were homogenized in 0.1M PCA solution by sonication and centrifuged at 17,400 *g* for 20 min at 4°C. The supernatant was injected onto the HPLC system. The mobile phase consisted of 50 mM citric acid, 0.1 mM EDTA, 8 mM KCl, 50 mM phosphoric acid, 100 mg/L octanesulfonic acid, and 6% methanol, pH adjusted to 3.85 by the addition of sodium hydroxide. The mobile phase was pumped at a flow rate of 0.2 mL/min by an Alexys LC100 pump connected via an Alexys AS100 autosampler to an Antec Leyden reverse phase analytical column (ALF-215 150 mm × 2.1 mm i.d.) maintained at 35°C. Neurotransmitter levels were detected using a glassy carbon flow cell (VT-03 Antec) with an ISAAC reference electrode. An external standard consisting of DA, NA, serotonin (5-HT), and metabolites in concentrations of 10^−7^, 0.5 × 10^−7^ and 10^−8^ M was injected at a volume of 4.0 µL for calibration. Samples were injected onto the column at 4.0 µL volumes, except for prelimbic and infralimbic samples which were injected at 8.0 µL due to the lower DA levels. Results were analysed using Alexys software data system. Bradford assay was used to adjust for protein content using the pellet remaining after sample centrifugation.

### Experiments 3A and 3B: Intra-NAc amphetamine infusions

#### Surgical procedures

Experiment 3A was conducted with a total of 104 rats and Experiment 3B with a total of 112 rats (mean weight 252 g, in the range 199–289 g). Both were run in two replications in which half the rats were surgically prepared for micro-injection in NAc core and the other half in NAc shell. To this end, rats underwent the same surgical procedure as in Experiment 2 except that bilateral stainless steel guide cannulae (22 gauge, length 11 mm below guide; Plastic One, Roanoke, VA, USA) were implanted to allow subsequent micro-injection (as described in the following) and aimed at the NAc: core at AP + 1.6 mm, ML ± 1.9 mm, DV −4.8 mm; shell at AP + 1.3 mm, ML ± 0.75 mm, DV −4.7 mm. Cannulae were held in place by dental cement and anchored to the skull with four fixing screws located on different bone plates. Removable obturators were inserted into the guide cannulae to prevent the cannulae from blocking.

#### Drug administration

In line with previous work (Joseph et al., 2000), a single sensitizing systemic injection of D-amphetamine sulphate (Sigma, Poole, UK) was administered (i.p.) 15 min prior to the pre-exposure stage. In Experiment 3A a dose of 1.0 mg/kg was used and in Experiment 3B a higher dose of 2.0 mg/kg was used. Control animals received an equivalent volume of saline.

The amphetamine micro-injections were administered prior to the conditioning stage. D-amphetamine sulphate (Sigma, Poole, UK) was dissolved in saline. In Experiment 3A a dose of 5.0 µg/side was used and in Experiment 3B this was increased to 10.0 µg/side (both doses expressed as the salt). Rats were lightly restrained, the dust caps and obturators were removed, and 31 gauge stainless steel infusion cannulae that protruded 2 mm beyond the tip of the guide cannulae were inserted into either the core or shell of the NAc. The infusion cannulae were connected to two 5 µL syringes mounted on an infusion pump. A volume of 0.5 µL per hemisphere was infused over 1 min and the infusion cannulae were left *in situ* for a further 1 min to allow absorption of the bolus. The infusion cannulae were then removed and the obturators and dust caps replaced. The animals were returned to the home cage before the onset of conditioning, 10 min after completion of the micro-injection. Control animals underwent the identical procedure but received infusions of saline.

#### Histological procedures

Following the completion of behavioural testing, rats received a lethal dose of sodium pentobarbitone. To aid verification of the placement of the cannulae tips, infusion cannulae were inserted and 0.5 µL Pontamine sky blue dye was infused following the microinfusion procedure described above. Thereafter the animals were decapitated with a guillotine. The headcaps and guide cannulae were removed and the brain taken out and fixed in formal saline for at least 7 days. Slices (80 µm thick) were made using a vibratome and were mounted onto gelatine-coated slides. Placement of the infusion cannulae tips was verified with a light microscope and the atlas of Paxinos and Watson (2005).

### Design and analysis

Statistical analysis was performed using analysis of variance (ANOVA) with alpha set at *p* < 0.05 for the rejection of the null hypothesis. Significant interactions were explored by simple effects analysis with further planned comparisons by *t*-test, where appropriate. Where necessary, raw latency data (time to first lick at pre-training and reshape) were log transformed so that their distribution was suitable for parametric analysis.

For the light tests, the between-subject factors were conditioning group (control, pre-exposed, overshadowed) and treatment. For the noise tests, the between-subject factors were conditioning group (control, overshadowing) and treatment. In Experiment 1, the treatment was drug (saline, amphetamine). In Experiment 2, the treatment was lesion (vehicle, core, shell). In Experiments 3A and 3B, the treatment was infusion (saline, amphetamine-core, amphetamine-shell). Replication was initially a factor in all analyses but was subsequently removed where there were no interactions with conditioning group or treatment.

## Results

### Experiment 1: Systemic amphetamine administration

#### Pre-training

ANOVA of the latency to first lick over the 10 days of pre-training showed no overall effect of drug (*F*_(1,65)_ = 1.75, *p* = 0.191) or conditioning group-to-be (*F* < 1), or any interaction between these factors (*F* < 1).

#### Reshape

ANOVA of latency to first lick yielded a main effect of conditioning group (*F*_(1,65)_ = 3.145, *p* < 0.05). This arose because the overshadowed group showed shorter latencies to complete the first lick compared to the pre-exposed group (*t*_(46)_ = 2.5, *p* < 0.05) and marginally longer latencies compared with the behavioural control group: mean log s (±SEM) control group = 1.253 (±0.15), pre-exposed group = 1.329 (±0.12) and overshadowed group = 0.888 (±0.13). However, latency to drink was unaffected by drug, as there was no effect of drug, nor any drug by conditioning group interaction (both *F* < 1).

#### Light test

**Figure 2.**
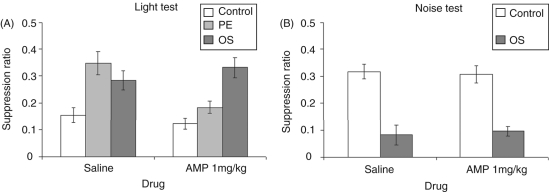
(A) Mean suppression ratio (±SEM) to the light for control (white bars), pre-exposed (light grey bars) and overshadowing (dark grey bars) groups following treatment with saline or 1.0 mg/kg amphetamine. (B) Mean suppression ratio (±SEM) to the noise for control (white bars) and overshadowing (dark grey bars) groups following treatment with saline or 1.0 mg/kg amphetamine.

ANOVA of the suppression ratios yielded an effect of conditioning group (*F*_(2,65)_ = 14.47, *p* < 0.001). However, as is clear from Figure 2A, the effects of conditioning were not equivalent across the drug groups. There was a trend towards higher levels of conditioning in the amphetamine-treated animals (*F*_(1,65)_ = 3.51, *p* = 0.066) and a conditioning group × drug interaction (*F*_(2,65)_ = 5.47, *p* < 0.01). Simple effects analysis of this interaction confirmed that amphetamine treatment was without effect in the control (*F* < 1) and overshadowed (*F*_(1,65)_ = 1.1, *p* = 0.304) groups. However, amphetamine clearly reduced LI in that pre-exposed rats showed marked suppression to the light compared with their saline-treated counterparts (*F*_(1,65)_ = 12.94, *p* < 0.001). Thus, with the experimental parameters adopted for the current study, amphetamine disrupted LI but was without effect on conditioning to the overshadowed light.

#### Noise test

All animals conditioned with the compound noise–light stimulus suppressed lick behaviour following onset of the noise stimulus and the unconditioned suppression to the noise measured in the behavioural control groups was unaffected by drug treatment (Figure 2B). ANOVA yielded an effect of conditioning group (*F*_(1,43)_ = 57.88, *p* < 0.001), but neither an effect of drug nor an interaction (both *F* < 1).

### Experiment 2: Shell and core 6-OHDA lesions

#### Neurochemical assay

Quantification of the selectivity of the Experiment 2 lesions by HPLC revealed that 11 out of the 71 6-OHDA-injected animals showed little evidence of DA depletion in either the core or shell NAc and consequently these animals were removed from the study. In total 12 further animals were assigned to their lesion group on the basis of the selectivity of the depletion produced rather than the injection coordinates. The criterion for assignment on the basis of the assay was less than 25% depletion in the intended core/shell region, coupled with relatively greater depletion in the adjacent shell/core region. On this basis, there were 35 animals in the shell group, 25 in the core group and 36 shams.

Table 1 shows the levels (pmol/µg brain tissue corrected for protein content) of DA, NA, and 5-HT in the five brain regions from which samples were taken as (A) absolute levels and (B) as the percentage depletion relative to sham levels. These results confirm that DA depletions in the shell group were selective to the medial shell NAc (−69%) and in this group there were no significant changes in DA levels in the core. Rats in the core group showed a statistically significant reduction in DA levels compared with vehicle-infused controls in both the core NAc (−61%) and in the medial shell NAc (−65%). Significant DA depletions were also found in both the prelimbic and infralimbic cortices, but not the caudate putamen. Desipramine pre-treatment successfully protected NA terminals in both subregions of NAc. There was some significant reduction in baseline NA levels in prelimbic cortex, but this change was unlikely to have been a direct effect of 6-OHDA injection in NAc. No significant changes in 5-HT were detected.

**Table 1.** (A) Levels of dopamine, noradrenalin and serotonin (pmoles per µg of protein content) of sham-, core- and shell-lesioned animals in core, shell, caudate putamen (CPu), prelimbic (PL) and infralimbic cortices (IL). (B) Percentage difference in dopamine, noradrenalin and serotonin levels of core- and shell-lesioned animals compared with vehicle-infused sham animals in the five brain regions assayed. *p < 0.05, *t*-test

(A)									
	Dopamine			Noradrenalin			Serotonin		
	Sham	Core lesion	Shell lesion	Sham	Core lesion	Shell lesion	Sham	Core lesion	Shell lesion
Core sample	4.652 (±0.391)	1.817 (±0.201)	4.563 (±0.401)	0.266 (±0.046)	0.273 (±0.098)	0.233 (±0.04)	0.289 (±0.018)	0.29 (±0.032)	0.301 (±0.058)
Shell sample	5.147 (±0.488)	2.088 (±0.476)	2.034 (0.199)	0.833 (±0.141)	0.841 (±0.203)	0.832 (±0.212)	0.5 (±0.051)	0.403 (±0.052)	0.464 (±0.051)
CPu sample	7.873 (±0.371)	7.271 (±0.438)	7.87 (±0.258)	0.165 (±0.009)	0.147 (±0.008)	0.151 (±0.006)	0.242 (±0.016)	0.218 (±0.01)	0.244 (±0.015)
PL sample	0.58 (±0.003)	0.25 (±0.003)	0.35 (±0.005)	0.21 (±0.01)	0.163 (±0.013)	0.143 (±0.011)	0.17 (±0.009)	0.159 (±0.012)	0.168 (±0.008)
IL sample	0.122 (±0.057)	0.045 (±0.007)	0.056 (±0.019)	0.379 (±0.099)	0.237 (±0.042)	0.196 (±0.038)	0.221 (±0.022)	0.185 (±0.026)	0.222 (±0.19)

(B)									
		Dopamine			Noradrenalin			Serotonin	
		Core lesion	Shell lesion		Core lesion	Shell lesion		Core lesion	Shell lesion
Core sample		−64.7%* (±3.7)	−1.2% (±11.1)		−13.5% (±23.2)	−13.6% (±12.4)		−4.4% (±10.1)	+1.2% (±14.4)
Shell sample		−65.4%* (±7.4)	−69.6%* (±3.2)		−13.6% (±14.7)	−16.4% (±10.5)		−18.5% (±6.4)	−1.4% (±8.1)
CPu sample		+1.7 (±5.1)	+2.9% (±4.3)		−10.7% (±5.8)	+0.3% (±5.6)		−3.8% (±4.9)	+4.5% (±4.6)
PL sample		−51.7%* (±5.7)	−41.2%* (±9.1)		−14.9%* (±5.9)	−30.1%* (±5.1)		−1.6% (±9.1)	+1.6% (±4.7)
IL sample		−34.9%* (±9.1)	−54.9%* (±6.2)		−19.4% (±9.8)	−32.7%* (±7.4)		−9.1% (±10.1)	−8.2% (±6.9)

#### Pre-training

ANOVA of the latency to first lick over the 5 days of pre-training showed no differences by conditioning group or lesion (maximum *F*_(8,348)_ = 1.41, *p* = 0.19).

#### Reshape

Table 2 displays the mean log (10) times (s) to complete the first lick in the reshape session following conditioning. The data show that the animals differed in the level of suppression seen to the box context with the longest latencies in the sham-lesioned control group compared with other lesion and conditioning groups. This observation was confirmed statistically as there was an interaction between conditioning group and lesion (*F*_(4,78)_ = 2.83, *p* < 0.05) but no main effect of conditioning group or lesion (maximum *F*_(2,78)_ = 1.22, *p* = 0.31). The interaction arose because there was an effect of conditioning group in the sham-lesioned rats (*F*_(2,87)_ = 3.76, *p* < 0.05), with shorter latencies in the pre-exposed (*t*_(20)_ = 3.1, *p* < 0.01) and overshadowed groups (*t*_(19)_ = 3.5, *p* < 0.01) compared with the controls (*p* < 0.05). However, there were no differences in latency to first lick between the three conditioning groups in either the core- or shell-injected rats (maximum *F*_(2,87)_ = 1.94, *p* = 0.15).

**Table 2.** Mean latency (log s) to complete the first lick on the reshape session for control, pre-exposed and overshadowed groups following sham or 6-hydroxydopamine lesions to either the core or shell subregions of the nucleus accumbens

	Control	Pre-exposed	Overshadowed
Sham	1.88 (±0.22)	1.30 (±0.17)	1.14 (±0.18)
Core	1.36 (±0.22)	0.94 (±0.29)	1.51 (±0.14)
Shell	1.21 (±0.18)	1.41 (±0.23)	1.35 (±0.16)

#### Light test

**Figure 3.**
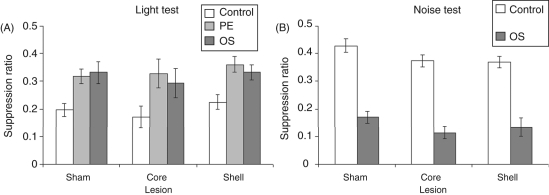
(A) Mean suppression ratio (±SEM) to the light for control (white bars), pre-exposed (light grey bars) and overshadowing (dark grey bars) groups following sham or 6-hydroxydopamine lesions to either the core or shell subregions of the nucleus accumbens. (B) Mean suppression ratio (±SEM) to the noise for control (white bars) and overshadowing (dark grey bars) groups following sham or 6-hydroxydopamine lesions to either the core or shell subregions of the nucleus accumbens.

The mean suppression ratios to the light CS are presented in Figure 3A. This shows that both LI and overshadowing were unaffected by lesion as all of the pre-exposed and overshadowed groups showed less conditioning to the light CS than the control animals, irrespective of lesion. This description of the data was supported statistically as ANOVA yielded an effect of conditioning group (*F*_(2,87)_ =15.28, *p* < 0.001) but neither an effect of lesion nor an interaction (maximum *F*_(2,87)_ = 1.1, *p* = 0.34).

#### Noise test

The mean suppression ratios to the noise CS are presented in Figure 3B. All animals that were conditioned with the compound (light + noise) CS showed marked suppression to the noise CS irrespective of lesion. As expected, the control animals showed little unconditioned suppression to the noise CS. ANOVA yielded an effect of conditioning group (*F*_(1,68)_ = 150.1, *p* < 0.001) and a marginal effect of lesion (*F*_(2,61)_ = 3.14, *p* = 0.051) but no interaction (*F* < 1). However, no further comparisons of the effect of lesion were statistically reliable. This means that the main effect of lesion arises because of overall increased suppression, without significant distinction between the effects of shell and core placements on conditioned and unconditioned suppression.

#### More stringent inclusion criterion

The above effects of lesion on reshape latencies and tone suppression provide a positive control in that the lesions were not simply ineffective, depleting DA insufficiently to have any behavioural effects. However, it remains possible that the lack of effect of the lesion on LI and overshadowing could potentially be because the 6-OHDA infusions did not produce sufficient DA cell loss. We therefore applied a more stringent inclusion criterion (i.e. minimum DA depletion of 50% in target region) and reanalysed the data accordingly. However, the reanalysis did not change the original conclusion that the lesions were without effect on either LI or overshadowing under the present experimental conditions. ANOVA again revealed no effect of lesion or conditioning group × lesion interaction (both *F* < 1).

### Experiments 3A and 3B: Intra-NAc amphetamine infusions

#### Histological verification

In Experiment 3A, 8 animals were excluded on the basis of histological verification leaving 47 core-implanted (30 infused with amphetamine and 17 with saline) and 49 shell-implanted animals (33 infused with amphetamine and 16 with saline). In Experiment 3B, 6 animals were excluded on the basis of histological verification leaving 56 core-implanted (37 infused with amphetamine and 19 with saline) and 50 shell-implanted animals (33 infused with amphetamine and 17 with saline).

**Figure 4.**
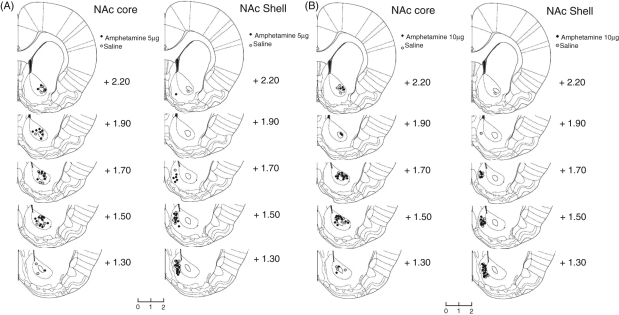
Histological assessment of cannula placements within the nucleus accumbens. Representative coronal sections from rats that received microinjections of 5.0 µg amphetamine (A) or 10.0 µg amphetamine (B) into the NAc core and NAc shell. Outlines are reproduced from Paxinos and Watson (1998) *The Rat Brain in Streretaxic Coordinates, 5th edition* with permission from Elsevier)and coordinates refer to the distance in millimetres anterior to bregma.

Figure 4 shows schematic reconstructions of infusion sites within the NAc core and NAc shell for amphetamine- and saline-infused animals. There was a clear anatomical dissociation in the location of the infusion sites between the medial shell and the core of the NAc.

#### Pre-training

In Experiment 3A, ANOVA of the latency to first lick over the 5 days of pre-training showed no differences by conditioning group or infusion (maximum *F*_(2,87)_ = 2.3, *p* = 0.11). Similarly, in Experiment 3B, there were no differences by conditioning group or infusion (maximum *F*_(2,96)_ = 1.4, *p* = 0.25).

#### Reshape

In neither experiment was there any evidence that the level of conditioning to the experimental chamber measured as latency to lick in the reshape sessions was affected by conditioning group or infusion (maximum *F*_(2,87)_ = 2.02, *p* = 0.14).

#### Light test

**Figure 5.**
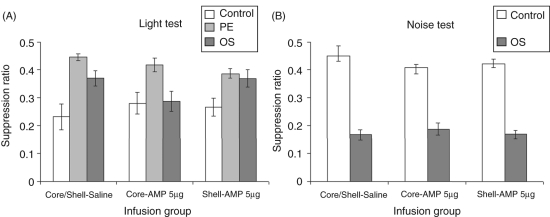
(A) Mean suppression ratio (±SEM) to the light for control (white bars), pre-exposed (light grey bars) and overshadowing (dark grey bars) groups following injection of vehicle or 5.0 µg amphetamine in either the core or shell subregions of the nucleus accumbens. (B) Mean suppression ratio (±SEM) to the noise for control (white bars), pre-exposed (light grey) and overshadowing (dark grey bars) groups following injection of vehicle or 5.0 µg amphetamine in either the core or shell subregions of the nucleus accumbens.

Figure 5A suggests that the groups differed by conditioning and infusion group in that the overshadowing effect appeared to be attenuated by infusions of 5 µg/side. However, this apparent reduction in overshadowing was not supported statistically. Analysis of the suppression ratio to the light CS revealed an effect of conditioning group (*F*_(2,87)_ = 18.82, *p* < 0.001) but no effect of infusion (*F* < 1) nor an interaction (*F*_(4,87)_ = 1.72, *p* = 0.15).

**Figure 6.**
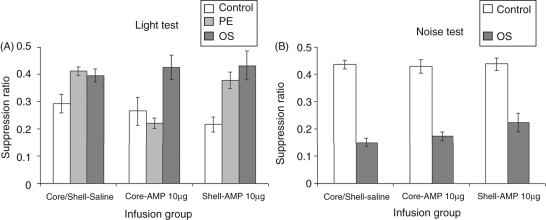
(A) Mean suppression ratio (±SEM) to the light for control (white bars), pre-exposed (light grey bars) and overshadowing (dark grey bars) groups following injection of vehicle or 10.0 µg amphetamine in either the core or shell subregions of the nucleus accumbens. (B) Mean suppression ratio (±SEM) to the noise for control (white bars), pre-exposed (light grey) and overshadowing (dark grey bars) groups following injection of vehicle or 10.0 µg amphetamine in either the core or shell subregions of the nucleus accumbens.

In Experiment 3B, there was no effect of conditioning group or infusion on the A periods (maximum *F*_(2,97)_ = 1.68, *p* = 0.19), confirming that the groups were again well matched for drinking prior to the critical suppression test. Figure 6A shows that there was no effect of amphetamine infusions into the shell on either LI or overshadowing at 10.0 µg/side. However, 10.0 µg/side amphetamine infused into the core appeared to abolish LI without affecting overshadowing. This description of the data was confirmed by ANOVA which yielded a marginal effect of infusion (*F*_(2,97)_ = 2.66, *p* = 0.075), a clear effect of conditioning group (*F*_(2,97)_ =14.27, *p* < 0.001) and moreover a significant interaction between these factors (*F*_(4,97)_ = 3.74, *p* < 0.01). Simple effects analysis of this interaction revealed no effect of infusion in either the control or overshadowed groups (both *F* < 1), but an effect of infusion in the pre-exposed animals (*F*_(2,97)_ = 9.33, *p* < 0.001) as the amphetamine-core LI group showed greater conditioning to the light compared with both their saline (*t*_(23)_ = 7.5, *p* < 0.001) and amphetamine-shell counterparts (*t*_(25)_ = 4.35, *p* < 0.001).

#### Noise test

There was no evidence that either dose of amphetamine had any effect on the level of suppression to the noise CS (maximum *F*_(2,67)_ = 1.46, *p* = 0.24). In both experiments (data shown in Figures 5B and 6B), overshadowed animals showed marked suppression to the noise CS (minimum *F*_(1,61)_ = 194.5, *p* < 0.001).

## Discussion

Experiment 1 confirmed that the LI aspect of the CER procedure was amphetamine-sensitive. However, despite that fact that the reduction in learning resulting from LI and overshadowing was (as in Experiments 2 and 3) near identical, the overshadowing aspect of the procedure was amphetamine-insensitive at the dose tested (1.0 mg/kg, i.p.). In Experiment 2, DA depletion produced by 6-OHDA in NAc was without a detectable effect on LI or overshadowing. However, at similar coordinates, and in the equivalent volume, the Experiment 3B amphetamine infusion in NAc core but not shell was demonstrated to abolish LI but not overshadowing, at the 10.0 but not the 5.0 µg/side dose. Thus, the effects of all three experimental manipulations on LI were in line with predictions, whilst there were no significant effects on overshadowing.

### The 6-OHDA lesions

In the present study, as a first step, the effects of DA depletion in shell and core sub-regions of NAc were tested with experimental parameters designed to produce reliable LI in the vehicle-injected controls, because these were the parameters suitable to test for the disruption of LI predicted to result from the amphetamine treatments (Weiner, 2003; Weiner and Arad, 2009) and to test for any disruption in overshadowing. That DA depletion in shell was without effect on LI conducted with these parameters suggests that reducing the actions of DA within NAc does not readily reproduce the pattern of results obtained with electrolytic and excitotoxic lesions to shell (Tai et al., 1995; Weiner, 2003; Weiner et al., 1996, 1999) and points to a particular role for DA within medial shell NAc in the modulation of LI. The present results are moreover consistent with *in vivo* studies of DA release in NAc showing that the expression of LI is associated with reduced DA release within the medial shell but not core NAc. Specifically, it has been shown that extracellular levels of DA are increased in the medial shell when a CS is paired with an aversive event but that this conditioned release is eliminated following non-reinforced pre-exposure to the CS (Jeanblanc et al., 2002; Murphy et al., 2001). Based on these studies, the results obtained with the 6-OHDA lesions are, at face value, entirely as expected.

However, lesion selectivity should be considered because complete NAc lesions that span both shell and core subregions are known to spare LI (Jongen-Relo et al., 2002; Konstandi and Kafetzopolous, 1993; Weiner et al., 1996). The 6-OHDA lesions tested in the present study were clearly different according to the placement of the 6-OHDA injection. Whilst the shell lesion was successfully selective in that the DA depletion produced did not extend to the core sample, injection at the ‘core’ placement depleted DA in shell and core. This pattern of anatomical selectivity of the shell but not core lesion is consistent with other reports of the effects of 6-OHDA infusions into the core and medial shell of the NAc (e.g., Sellings and Clarke, 2003; Sellings et al., 2008; Sokolowski and Salamone 1998) and may relate to the asymmetric connectivity between shell and core (van Dongen et al., 2005). In any event, the shell lesion, the site at which electrolytic and excitotoxic lesions abolish LI, was both neuroanatomically and neurochemically selective. The lack of effect of the present shell lesions could be suggested to be due to the procedure used to differentiate shell and core in the present study, principally by varying the laterality of injection, in order to lesion shell without passing through overlying core NAc. However, the ventral aspect of shell is similarly intact in other studies which show LI abolition after shell but not core lesions (Schiller et al., 2006; Weiner 2003; Weiner et al., 1996, 1999).

The significant changes in prelimbic and infralimbic cortices could be secondary, consistent with the known interconnectivity of prelimbic and infralimbic regions with NAc core and shell, respectively (Berendse et al., 1992; Gorelova and Yang, 1997). Alternatively, these changes could be a direct consequence of damage to dopaminergic fibres en route to prefrontal cortex, which pass near NAc. However, there was no evidence that the low volume of neurotoxin injected in the present study spread beyond NAc: there were no significant neurochemical changes dorsally in caudate putamen.

Overall, the magnitude of the DA depletions produced by the local injections of 6-OHDA in the present study was not very large and this may account for their lack of effect. It is not uncommon to have 75–90% depletions of DA with local injections of 6-OHDA (e.g., Correa et al., 2002; Cousins et al., 1993; Salamone et al., 2001; Sokolowski and Salamone 1998). However, the exclusion criterion adopted made no difference to the lack of effect on the learning measure and the lesions were nonetheless behaviourally effective in that they moderated the level of contextual conditioning to the box cues measured at reshape. Similarly, there was some evidence that the lesions affected (conditioned and unconditioned) suppression to the noise CS. In other tests (of novel object recognition) these lesions showed dissociable behavioural effects (Nelson et al., 2010). Subsequently using a reduced number of pre-exposures, LI enhancement has been demonstrated with similar levels of DA depletion in shell (Nelson et al., 2009), and as would be predicted on the basis of the available vivo dialysis and voltammetry studies (Jeanblanc et al., 2002; Murphy et al., 2001); see also the discussion above.

### Amphetamine abolition of LI mediated in core

Conditioning is known to be the critical experimental stage at which amphetamine effects on LI are mediated (Joseph et al., 2000; Weiner et al., 1988) and in the present study micro-injection in core but not shell NAc disrupted LI at the conditioning stage of the procedure. In contrast, electrolytic and excitotoxic core lesions enhance rather than disrupt LI (Schiller et al., 2006; Weiner 2003; Weiner et al., 1996, 1999). Thus, the current findings further suggest that amphetamine disrupts LI by activating the core and promoting behavioural switching to the stimulus-reinforcement contingencies acquired at conditioning. This is consistent with the switching hypothesis of LI which posits that disrupted LI is the result of excessive switching and that switching is subserved by a mechanism that resides in the NAc core and is activated by increased DA levels in core (Weiner, 2003). Moreover, if such excitatory effects of amphetamine reflect a DA D1 rather than a D2 profile of action (Greengard, 2001; Traynor and Neubig, 2005), this would in turn suggest DA D1-mediation of the abolition of LI, at least in core NAc.

Experiments 3A and 3B also show the importance of dose of micro-infusion and sensitizing injection. Amphetamine injected in NAc at 5.0 µg/side was sufficient to abolish LI in an earlier study (Joseph et al., 2000), although at a higher injection volume of 1 µL, but was without effect on LI under our experimental conditions. Experiment 3B used a higher dose of 10.0 µg/side, also standard in studies of this kind (Ellenbroek et al., 1997; Killcross and Robbins, 1993). However, neither of these previous studies showed abolished LI after amphetamine injection in NAc at 10.0 µg/side. Thus, the emergence of the effect on LI in Experiment 3B may in part be attributable to the increased dose of sensitizing injection required under our experimental conditions (2.0 mg/kg). In any event, our results further underscore the importance of such an injection (see Joseph et al., 2000): earlier studies which failed to show any effect on LI of amphetamine when directly injected into NAc did not use a sensitizing injection (Killcross and Robbins, 1993) and/or did not target core NAc (Ellenbroek et al., 1997).

### Why were there no effects on overshadowing?

The 1.0 mg/kg D-amphetamine dose used in Experiment 1 is equivalent to that found to abolish LI in CER procedures (Weiner et al., 1981, 1984, 1987, 1988; Killcross et al., 1994) and has previously been reported to abolish overshadowing in CER procedures (O’Tuathaigh and Moran, 2002, 2004; O’Tuathaigh et al., 2003; but see Horsley and Cassaday, 2003). The different strains of rats used may account for this difference between laboratories. The nonsignificant results of Experiment 3A in which the overshadowing effect showed a tendency to be reduced after infusions of 5µg/side suggests that effects on overshadowing might be demonstrated in lower dose ranges under our experimental conditions (Figure 5A).

## Conclusions

The present results suggest that lesion-induced abolition of LI is not readily reproduced by regionally restricted DA depletion within NAc and that core rather than shell NAc mediates amphetamine-induced abolition of LI. The lack of effect after the 6-OHDA core lesion was as expected, based on all lesion studies to date. The lack of effect of the 6-OHDA shell lesion, whilst apparently contrary to the effects of excitotoxic and electrolytic lesions, was as expected based on dialysis and voltammetry studies.

Amphetamine treatments were without significant effect on overshadowing in the present study, possibly in relation to dose, and pointing to dissociation in the neuromodulatory mechanisms for salience modulation as measured in LI and overshadowing procedures. Further studies will be needed to determine the contributions of the DA D1- and D2-like receptor families in the abolition and enhancement of LI and overshadowing.
